# Supplementary material for: Inclusion Complexes of 3,4-Ethylenedioxythiophene with Per-Modified β- and γ-Cyclodextrins
Source: Molecules. 2023 Apr 12;28(8):3404. doi: 10.3390/molecules28083404 (PMC10143540; doi:10.3390/molecules28083404)
Supplement: Supplementary file 1 [file molecules-28-03404-s001.zip › molecules-2355882-supplementary.pdf]

# Inclusion Complexes of 3,4-Ethylenedioxythiophene with Per-Modified β- and γ-Cyclodextrins

**Aurica Farcas *, Ana-Maria Resmerita, Mihaela Balan-Porcarasu, Corneliu Cojocaru, Cristian Peptu and Ion Sava ***

''Petru Poni'' Institute of Macromolecular Chemistry, 700487 Iasi, Romania
* Correspondence: afarcas@icmpp.ro (A.F.); isava@icmpp.ro (I.S.)

## 1. Characterization data of compounds

1.1. $^{1}$H-NMR and $^{13}$C-NMR of TMe-βCD and TMe-γCD in $D_2O$

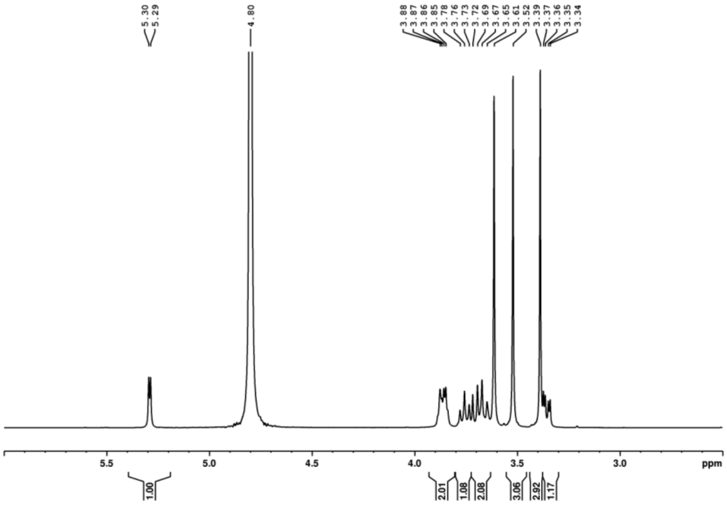


**Figure S1.** $^{1}$H-NMR spectrum of TMe-βCD in $D_2O$.

$^{1}$H-NMR ($D_2O$, 400 MHz), δ (ppm): 5.29 (d, *J* = 3.5 Hz, 7H, H1), 3.88–3.85 (m, 14H, H5, H6a), 3.76 (t, *J* = 8.9 Hz, 7H, H4), 3.72–3.65 (m, 14H, H3, H6b), 3.61 (s, 21H, C2-$OCH_3$), 3.52 (s, 21H, C3-$OCH_3$), 3.39 (s, 21H, C6-$OCH_3$), 3.36 (dd, *J* = 3.5 Hz, *J* = 9.6 Hz, 7H, H2).

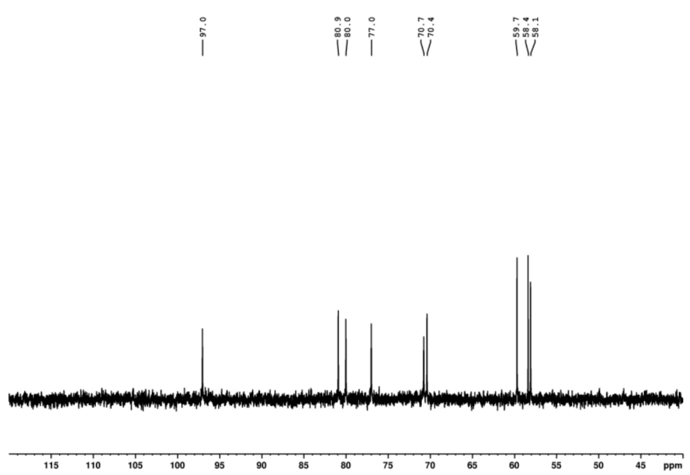

**Figure S2**. $^{13}$C-NMR spectrum of TMe-βCD in $D_2O$.

$^{13}$C-NMR ($D_2O$, 100 MHz), δ (ppm): 97.0 (C1), 80.9 (C3), 80.0 (C2), 77.0 (C4), 70.7 (C6), 70.4 (C5), 57.9 (C2-$OCH_3$), 58.4 (C6-$OCH_3$), 58.1 (C3-$OCH_3$).

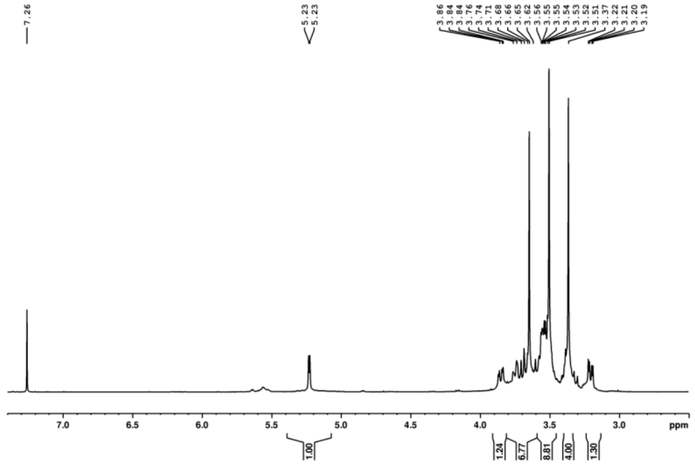

**Figure S3.** $^{1}$H-NMR spectrum of TMe-γCD in $CDCl_3$.

$^{1}$H-NMR ($CDCl_3$, 400 MHz), δ (ppm): 5.23 (d, *J* = 3.6 Hz, 8H, H1), 3.85 (dd, *J* = 10.7 Hz, *J* = 3.2 Hz, 8H, H6a), 3.76–3.62 (m, H3, H5, C2-$OCH_3$), 3.56–3.51 (m, H4, C3-$OCH_3$), 3.37 (s, 24H, C6-$OCH_3$), 3.21 (dd, *J* = 3.6 Hz, *J* = 9.7 Hz, 8H, H2).

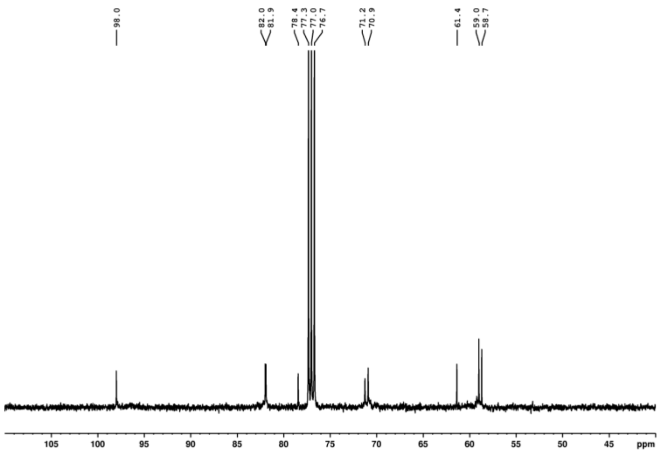


**Figure S4**. $^{13}$C-NMR spectrum of TMe-γCD in $CDCl_3$.

$^{13}$C-NMR ($CDCl_3$, 100 MHz), δ (ppm): 98.0 (C1), 82.0 (C3), 81.9 (C2), 78.4 (C4), 71.2 (C6), 70.9 (C5), 61.4 (C2-$OCH_3$), 59.0 (C6-$OCH_3$), 58.7 (C3-$OCH_3$).

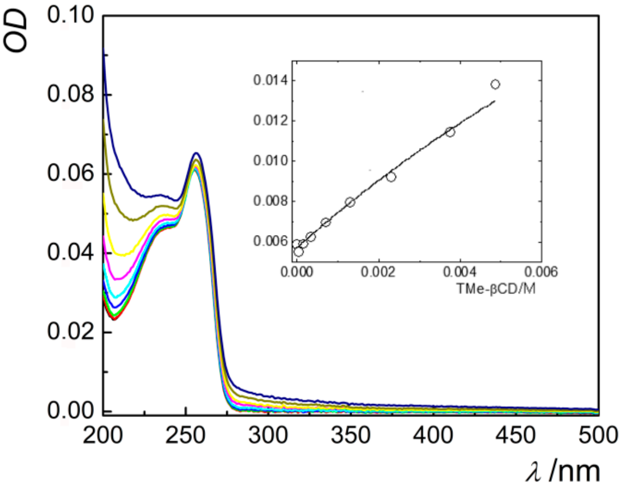


**Figure S5.** Changes in the absorption spectra of the monomer EDOT upon addition of increasing amounts of TMe-βCD in water. The fitted binding constant curve (according to a 1:1 host-guest complexation stoichiometry) is shown in the inset.

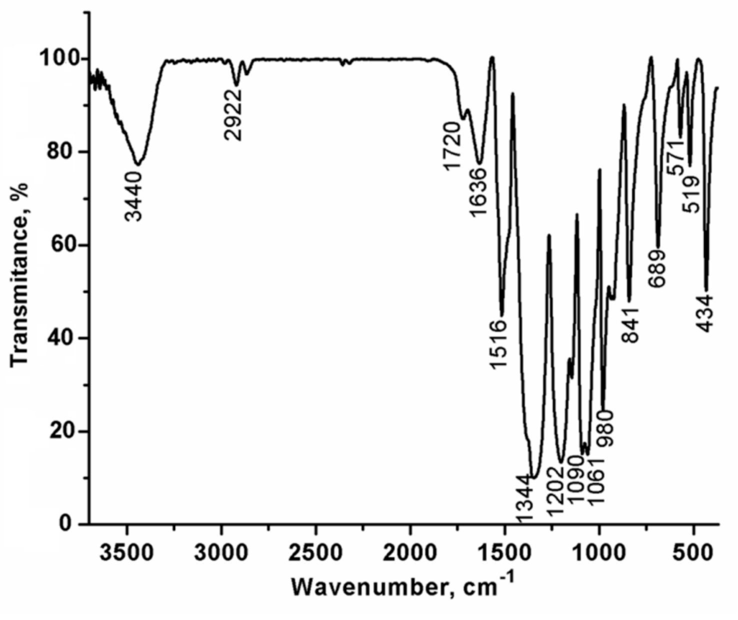


**Figure S6.** FT-IR spectrum of EDOT·TMe-βCD.

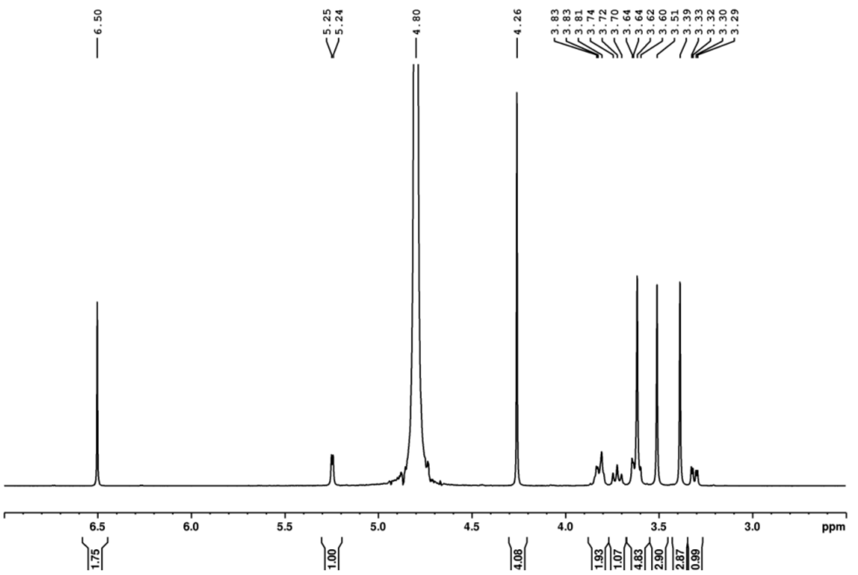


**Figure S7.** $^{1}$H-NMR spectrum of EDOT·TMe-βCD in $D_2O$.

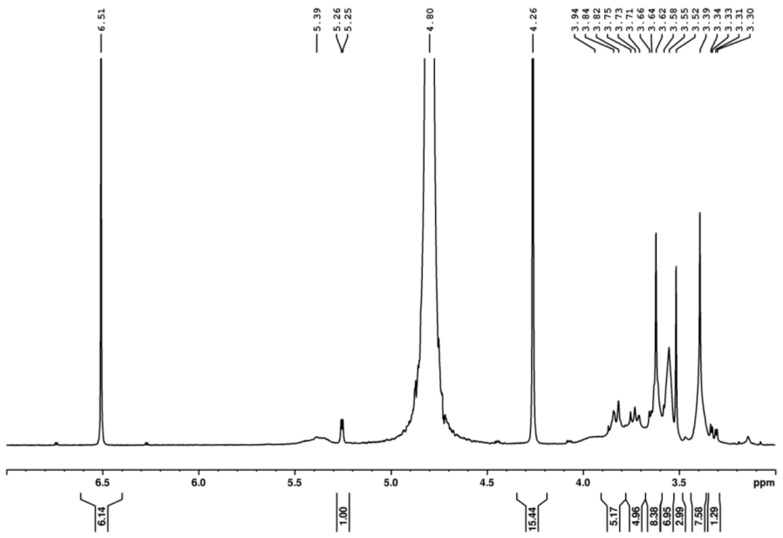

**Figure S8.** $^{1}H$-NMR spectrum of EDOT·TMe-γCD in $D_2O$.

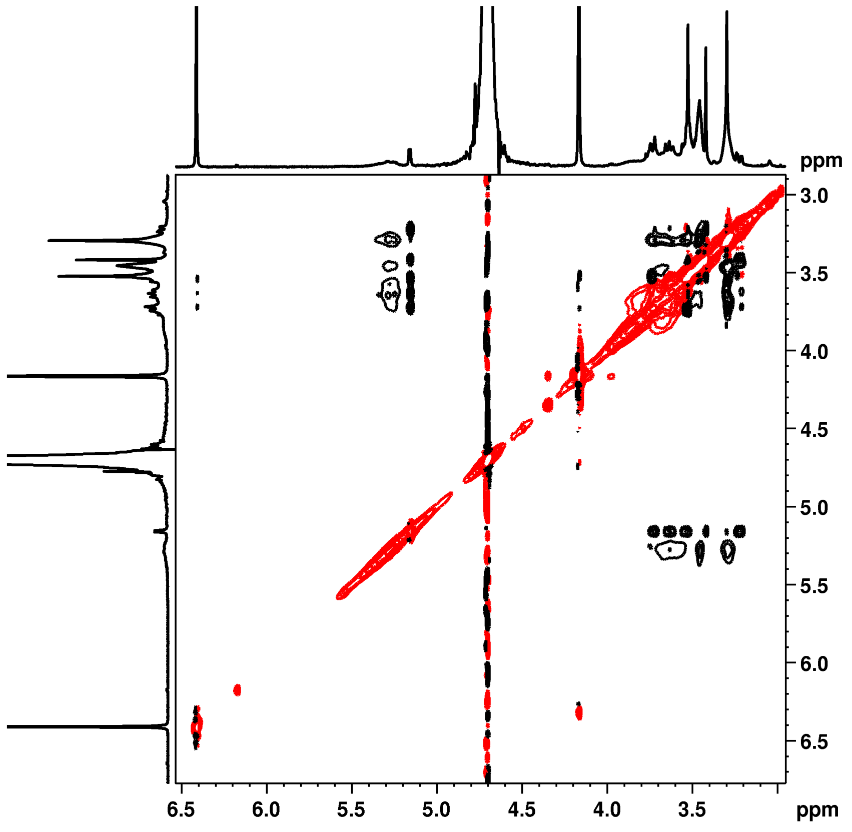

**Figure S9**. H-H ROESY (400 MHz, $D_2O$) spectrum for EDOT·TMe-γCD.

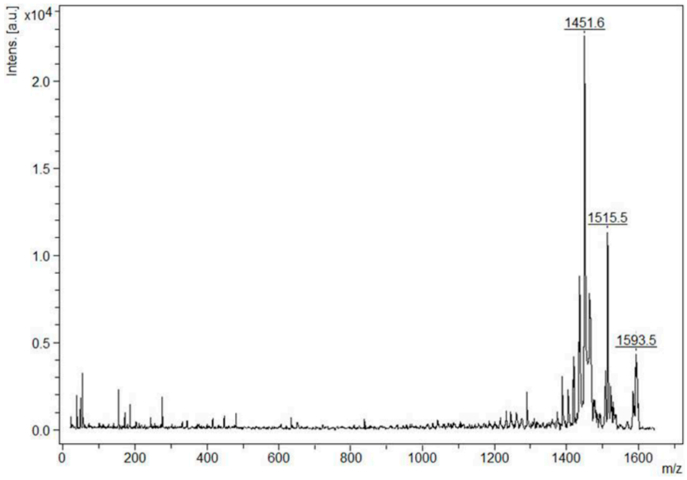


**Figure S10**. The positive-ion reflectron MALDI-TOF MS spectrum of the EDOT·TMe-βCD complex.

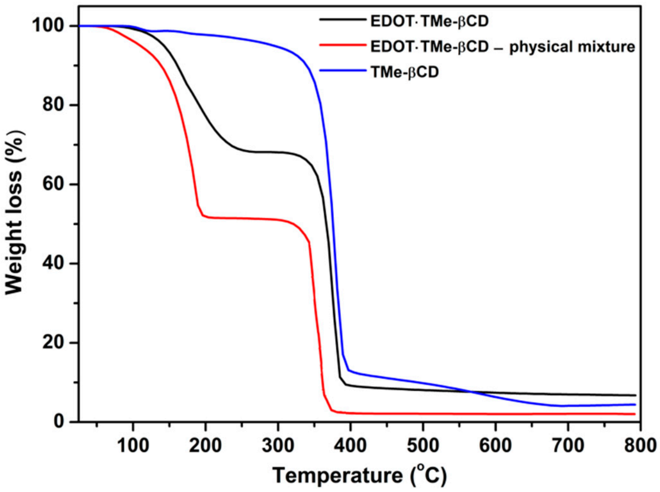


**Figure S11**. Thermograms of the EDOT·TMe-βCD inclusion complex (black line), physical mixture (red line) and TMe-βCD (blue line).
